# Depressive symptoms among Peruvian adult residents amidst a National Lockdown during the COVID-19 pandemic

**DOI:** 10.1186/s12888-021-03107-3

**Published:** 2021-02-18

**Authors:** Daniel A. Antiporta, Yuri L. Cutipé, Maria Mendoza, David D. Celentano, Elizabeth A. Stuart, Andrea Bruni

**Affiliations:** 1 Department of Epidemiology, Johns Hopkins Bloomberg School of Public Health, Baltimore, MD USA; 2 Mental Health Direction, Ministry of Health, Lima, Peru; 3 Department of Mental Health, Johns Hopkins Bloomberg School of Public Health, Baltimore, MD USA; 4 Pan American Health Organization/World Health Organization, Lima, Peru

## Abstract

**Background:**

Population health and well-being in Latin America, the current epicenter of the COVID-19 pandemic, has been severely affected during the past semester. Despite the growing evidence about the link between the pandemic, its control measures, and mental health worldwide, there is still no regional evidence of the potential mental health impact. We describe the prevalence and distribution of depressive symptoms across demographic and socioeconomic risk factors in the Peruvian population amidst a national lockdown during the COVID-19 pandemic.

**Methods:**

Cross-sectional study conducted during the community transmission phase and national lockdown in Peru (May 4th–16th, 2020). We recorded 64,493 responses from adult Peruvian residents through an opt-in online questionnaire. All analyses were weighted using raking based on proportions of sociodemographic variables from the last Peruvian census in 2017. The prevalence of depressive symptoms was calculated using the Patient Health Questionnaire (PHQ-9) score of 10 or more. We identified associated demographic and socioeconomic factors by prior mental health diagnosis. Sensitivity analysis considered an alternative cut-off point for depressive symptoms of PHQ-9 ≥ 14.

**Results:**

A total of 57,446 participants were included in the analytical sample. A third of the participants (*n* = 23,526, unweighted) showed depressive symptoms in the 2 weeks prior to the study. Participants who reported a previous mental health diagnosis doubled the sample prevalence of depressive symptoms (59, 95%CI 56.7, 61.4%) of those without a prior diagnosis. Psychosocial and functioning reactions were largely more prevalent among females and young adults. A dose-response relationship was found between household income and depressive symptoms across previous mental health diagnosis strata, being as low as 32% less in the wealthiest than the most impoverished group (PR: 0.68, 95%CI 0.58,0.79). Other critical factors associated with a higher burden of depressive symptoms were lower education level, single, unemployed, and chronic comorbidity.

**Conclusions:**

An increased burden of depressive symptoms and psychosocial reactions has emerged during the COVID-19 pandemic in Peru compared to previous years. The mental health burden disproportionately affects women, the younger population, and those with low income and education. As the country eases the social distancing measures, it is crucial to use local evidence to adjust public health policies and mental health services to the renewed population needs.

**Supplementary Information:**

The online version contains supplementary material available at 10.1186/s12888-021-03107-3.

## Introduction

The COVID-19 pandemic has substantially impacted health and well-being worldwide. Particular interest has been given to the detriments on mental health due to the disease spread, infection status, and policies to control the pandemic [1]. Although academic circles and mass media have highlighted the critical role of mental health [2], empirical data is limited, and international guidelines are based mainly on expert recommendations [3]. National health bodies, especially those in high transmission settings, require timely data to plan and implement evidence-based policies to protect mental health during the present crisis.

Severe SARS-Cov-2 infections could profoundly impact psychological well-being inducing mental conditions, including fatigue and delirium [4]. Recent evidence points out adverse neurological effects related to severe illness such as encephalopathies, inflammatory central nervous system syndromes, etc. [5]. Besides the direct biological effects of the infection on mental health, public policies, including social distancing and lockdowns, could also increase the burden of psychological reactions due to isolation, domestic violence, and the use of psychoactive substances [6, 7]. Mental health research needs to estimate the disease burden associated with the pandemic and quantify attendant mental health problems.

The Americas have become the current epicenter of the pandemic [8], mounting 31 million cases and 786,808 deaths as of December 16th [9] Forecasts suggest a sharp increase during the third quarter of 2020, affecting countries like Brazil, Chile, and Peru [10]. Countries’ responses to the pandemic in South America have included not only social distancing measures and testing but also mental health support interventions through hotlines, virtual screenings, and telemedicine [11]. Despite these laudable efforts, the effect of the pandemic and control policies on the public’s mental health and mitigation of the further spread of COVID-19 is unknown, which challenges the elaboration of national plans and evaluation of these actions.

Risk stratification is crucial to bring adequate and timely psychological support to specific populations. Early data revealed an increasing burden of psychosocial reactions in first respondents, such as emergency room (ER) and intensive care unit (ICU) health professionals [12], findings common in other crises. Nonetheless, the general population has also shown increasing stress and different psychosocial reactions during lockdowns and highly transmission contexts. For instance, in China, youth showed high rates of anxiety and depression disorder [13], while school-aged children who remained at home exhibited depressive symptoms [14].

The current scenario demands innovative research methods such as mobile or web-based tools to collect critical information rapidly during the pandemic [15]. However, awareness has been raised around the challenges of using digital tools to collect data [16, 17]. These tools can facilitate the epidemiological response from symptom screening, contact tracing, and quarantine measures.

This study aimed to identify and describe the distribution of depressive symptoms’ using a web-based questionnaire in the Peruvian population during the national lockdown due to the pandemic. Moreover, we identified associated factors and their magnitude to compare them with regional and global estimates. We report major findings by key strata of geographical location, sex, age, and previous mental health diagnosis.

## Methods

### Study design and settings

We conducted a cross-sectional study using an opt-in online anonymous questionnaire distributed on social media by key partners: the Peruvian Ministry of Health (MoH) and the Pan American Health Organization Peru country office. Eligible participants were 18 years of age or older, of any nationality, living in Peru, and with access to an internet connection. Supplementary files 1 & 2 show the English and Original versions of the survey.

### Sample characteristics

**Fig. 1:**
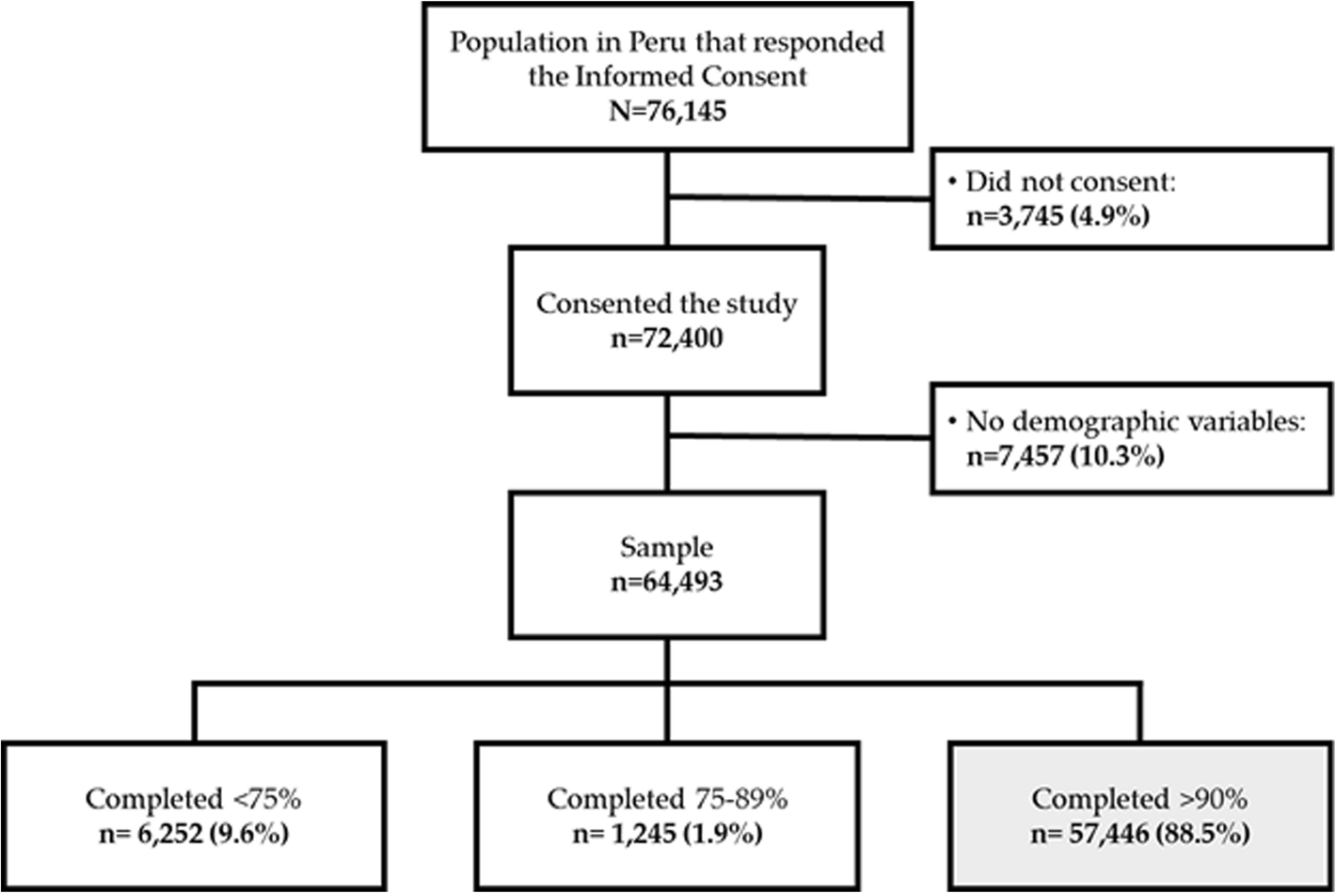
Flowchart for the study and analytical sample

In our final sample, we included all participants who consented to participate, provided sociodemographic variables such as sex, age, education, and geographical location, and completed at least 90% of the questionnaire. The 90% progress level corresponded to the survey’s last question about the average monthly household income, already recorded as a categorical variable in a previous question. After completing the selection process, only one response was flagged as spam, and after careful consideration, was included in the final sample. The flowchart in Fig. 1 shows the selection of the analytical sample.

### Procedures

The questionnaire was released on PAHO’s and MoH’s social media accounts, including Twitter and Facebook, on May 4th, almost 6 weeks after the start of the country lockdown. At the moment of the survey launch, Peru was facing the community transmission phase of the pandemic with up to 47,372 COVID-19 cases. Access to start the questionnaire was available for 7 days (May 4th to 11th), and participants had 7 days to complete it since the start date (up to May 18th). We used Qualtrics to design and collect the respondents’ data, who needed to validate a captcha code to access the informed consent.

The questionnaire included questions about demographic characteristics, mental health, COVID-19 infection, symptoms, and socioeconomic information. Mental health questions mostly referred to the past 2 weeks before completing the questionnaire. These questions included the previous diagnosis of a mental health problem, a sequential testing approach using the PHQ-2 and PHQ-9, and the WHO-UNCHR Assessment Schedule of Serious Symptoms in Humanitarian Settings (WASSS) [18].

The PHQ-9 is a standardized screening tool that includes nine criteria for major depression. It has been validated in the Peruvian population [19, 20] and has been used in the national Demographic Health Survey (DHS) in Peru since 2014. Sequential testing, presenting participants, who scored at least 2 points in the PHQ-2, the other seven questions of the PHQ-9 has reported being pragmatic, preserving classification accuracy, and reducing response burden [21–23]. We used the Spanish version of both tools, PHQ-9 and WASSS, from the Peruvian DHS and the WHO official publication, respectively.

At the end of the questionnaire, all respondents were shown information about available mental health hotlines and local community health centers.

### Outcomes

The primary outcome for this study was the prevalence of depressive symptoms (PHQ-9 ≥ 10 points, major depressive episode) [24]. Respondents that scored less than 2 points in the PHQ-2 were included and were assigned a value less than 10 points. Selected secondary outcomes were anhedonia (PHQ-9), suicidal ideation (PHQ-9), and impaired functioning (WASSS) reported using a Likert scale.

We also explored the proportion of respondents who reported having a mental health symptom at least 7 days or more in the past 2 weeks, using items from the PHQ-9 and part A of WASSS by key strata. Supplementary file 3 shows the questions and outcomes in the PHQ-9 and WASSS.

### Covariates

We selected four key strata to report the study outcomes: sex, age group, geographical location, and self-reported previous diagnosis of a mental health problem. The latter was a binary variable assessed by the question, “*Have you ever been diagnosed with any mental health problem?*”. Geographical location was a categorical variable with four levels: Metropolitan Lima (Lima Province and Callao), Coastal (the coastline of Peru), Andean (the Andean mountainous region), and Amazonian (the Amazonia forest) regions, following the methodology from the Peruvian DHS.

We included demographic and socioeconomic information as potential associated factors: education level, household size, employment status, household income, and marital status. Health characteristics were also included, such as the presence of any key comorbidities (obesity, hypertension, cardiovascular disease, cancer, diabetes, and immune disease) and COVID-19 infection history (yes/no).

### Statistical analysis

We estimated individual weights using raking for all analytical sample observations based on key demographic variables: sex, age, education level, and location to emulate the Peruvian population distribution. Raking used marginal proportions for each variable from the latest Peruvian national census from 2017 [25]; see Supplementary file 4 for details regarding raking values. All analyses presented in this study used raking weights to calculate proportions and regression estimates.

We explored the distribution of PHQ-9 and WASSS items prevalence by geographical location, age group, sex, and prior mental health condition and tested for differences across them. Multivariate analyses were performed to identify associated factors for the primary outcome and selected secondary outcomes by prior mental health condition status (yes, no, not responded). We used a series of sex-age adjusted generalized linear models to individually test the association of sociodemographic and health variables, using a Poisson distribution with robust standard errors for the primary outcome and multinomial logistic regressions for secondary outcomes.

Sensitivity analyses to test the robustness of our findings included using (1) subsets of data, including those who had 100 and 75% of survey completion, and (2) an alternative PHQ-9 cut-off point for depressive symptoms. Regarding the latter, recent evidence showed that the 10-point cut-off could overestimate the burden of moderate to severe depressive symptoms; we evaluated a recommendation of a threshold of ≥14 [26].

## Results

The eligible sample comprised data from 57,446 respondents who were predominantly young adults (72%), female (66%), living in the capital province (53%), and completed undergraduate degrees (47%). Marginal proportions for key sociodemographic variables corresponded to the values from the Peruvian Census 2017 after raking; see Supplementary file 4.

Weighted results showed that one-quarter of the sample was unemployed, and a similar proportion reported receiving assistance from the Government during the lockdown. Nearly half of respondents had a monthly household income of less than 1860 PEN (547 USD), although 28% of the sample preferred not to respond. Differences across regions were more evident regarding education level and household income. Table 1 summarizes the key characteristics of the participants by geographical location.

**Table 1:** Selected sociodemographic and health characteristics across geographical regions, *n* = 57,446^a^

	Geographical Location				
	Metropolitan Lima	Rest of Coast	Andes	Jungle	Total
	*n* = 32,927	*n* = 11,778	*n* = 10,762	*n* = 1979	n = 57,446
	n (%)	n (%)	n (%)	n (%)	n (%)
**Female**	22,710 (55.3%)	7633 (51.7%)	6573 (48.7%)	1144 (46.4%)	38,060 (51.5%)
**Age group**					
18–24 years	10,261 (9.7%)	4418 (17.4%)	4193 (23.7%)	691 (22.9%)	19,563 (17.3%)
25–34 years	12,254 (16.8%)	4393 (24.8%)	4051 (26.4%)	821 (30.5%)	21,519 (23.0%)
35–55 years	6282 (19.5%)	1927 (20.5%)	1683 (20.3%)	310 (21.4%)	10,202 (20.2%)
45–54 years	2664 (17.1%)	674 (15.1%)	571 (15.5%)	107 (14.6%)	4016 (15.9%)
55 years+	1466 (36.8%)	366 (22.1%)	264 (14.0%)	50 (10.6%)	2146 (23.6%)
**Education**					
Less than high school	1250 (30.3%)	547 (31.1%)	362 (18.7%)	88 (18.6%)	2247 (25.7%)
Completed high school	16,254 (49.3%)	5752 (46.8%)	5292 (55.0%)	873 (52.6%)	28,171 (50.8%)
Undergraduate or higher	15,423 (20.4%)	5479 (22.2%)	5108 (26.4%)	1018 (28.8%)	27,028 (23.5%)
**Marital status**					
Single	19,476 (34.6%)	6817 (37.6%)	6656 (46.1%)	1109 (44.1%)	34,058 (39.9%)
Married	5604 (30.2%)	1879 (27.1%)	1516 (22.2%)	285 (21.0%)	9284 (26.1%)
Living together	6512 (22.6%)	2728 (27.6%)	2261 (24.8%)	544 (30.8%)	12,045 (25.2%)
Other	1335 (12.6%)	354 (7.6%)	329 (6.9%)	41 (4.1%)	2059 (8.8%)
**Household size ≥ 4**	19,884 (57.2%)	7312 (60.1%)	6177 (54.6%)	1109 (55.6%)	34,482 (56.9%)
**Employment (2118 missing)**					
Formal employment	11,868 (28.4%)	3326 (25.6%)	2735 (25.2%)	672 (32.1%)	18,601 (32.1%)
Informal employment	3477 (14.9%)	1466 (17.1%)	1468 (16.7%)	274 (15.1%)	6685 (15.1%)
Unemployed before lockdown	7150 (20.6%)	2608 (17.1%)	2451 (18.4%)	350 (16.5%)	12,559 (16.5%)
Recently unemployed	10,432 (36.1%)	4378 (40.2%)	4108 (39.7%)	683 (36.4%)	19,601 (36.4%)
**Received any help from Government**	7082 (24.9%)	3208 (29.2%)	2304 (22.4%)	583 (29.5%)	13,177 (25.5%)
**Household income (195 missing)**					
Up to 930 PEN	8651 (30.1%)	4049 (38.2%)	3497 (34.3%)	633 (33.8%)	16,830 (33.8%)
931–1860 PEN	6770 (19.9%)	2451 (17.7%)	2096 (17.8%)	373 (17.2%)	11,690 (18.4%)
1861–2790 PEN	3219 (8.3%)	1047 (6.4%)	1067 (9.1%)	228 (9.9%)	5561 (8.2%)
2791–4650 PEN	3061 (6.9%)	751 (5.5%)	764 (6.2%)	151 (6.9%)	4727 (6.3%)
More than 4651 PEN	3846 (7.9%)	538 (3.9%)	510 (4.1%)	96 (3.9%)	4990 (5.4%)
Prefer not to respond	7261 (26.9%)	2907 (28.4%)	2796 (28.5%)	489 (28.3%)	13,453 (27.9%)
**COVID-19 infection (136 missing)**	298 (1.4%)	82 (0.9%)	36 (0.4%)	42 (2.5%)	458 (1.0%)
**Comorbidities (3158 missing)**	8423 (41.4%)	3038 (36.0%)	2394 (31.8%)	509 (34.3%)	14,364 (36.4%)
**Previous Mental Health Condition**					
No	26,084 (82.1%)	10,115 (85.0%)	8813 (82.7%)	1716 (86.5%)	46,728 (83.3%)
Yes	6120 (16.3%)	1423 (13.3%)	1644 (14.6%)	233 (12.5%)	9420 (14.7%)
Not responded	723 (1.6%)	240 (1.7%)	305 (2.8%)	30 (1.0%)	1298 (2.0%)
**PHQ-2 score ≥ 2 (196 missing)**	22,570 (60.7%)	7618 (59.1%)	7513 (64.5%)	1242 (59.7%)	38,943 (61.5%)
**Depressive Symptoms (196 missing)**	13,736 (33.8%)	4437 (32.9%)	4659 (38.0%)	694 (32.7%)	23,526 (34.9%)

Table presents n (%) where *n* is unweighted counts and *%* correspond to weighted proportions

^a^Unweighted observations

Respondents with a previous mental health diagnosis were more prevalent in the Capital City (16%) and lowest in the Amazon (13%). These geographical patterns were similar for those who sought mental health care and had at least one contact with the mental health system within 2 weeks before the survey. Around half of those who sought help managed to contact the health system; however, differences persisted by region, detrimental to Amazon residents.

### Mental health symptoms and reactions

**Fig. 2:**
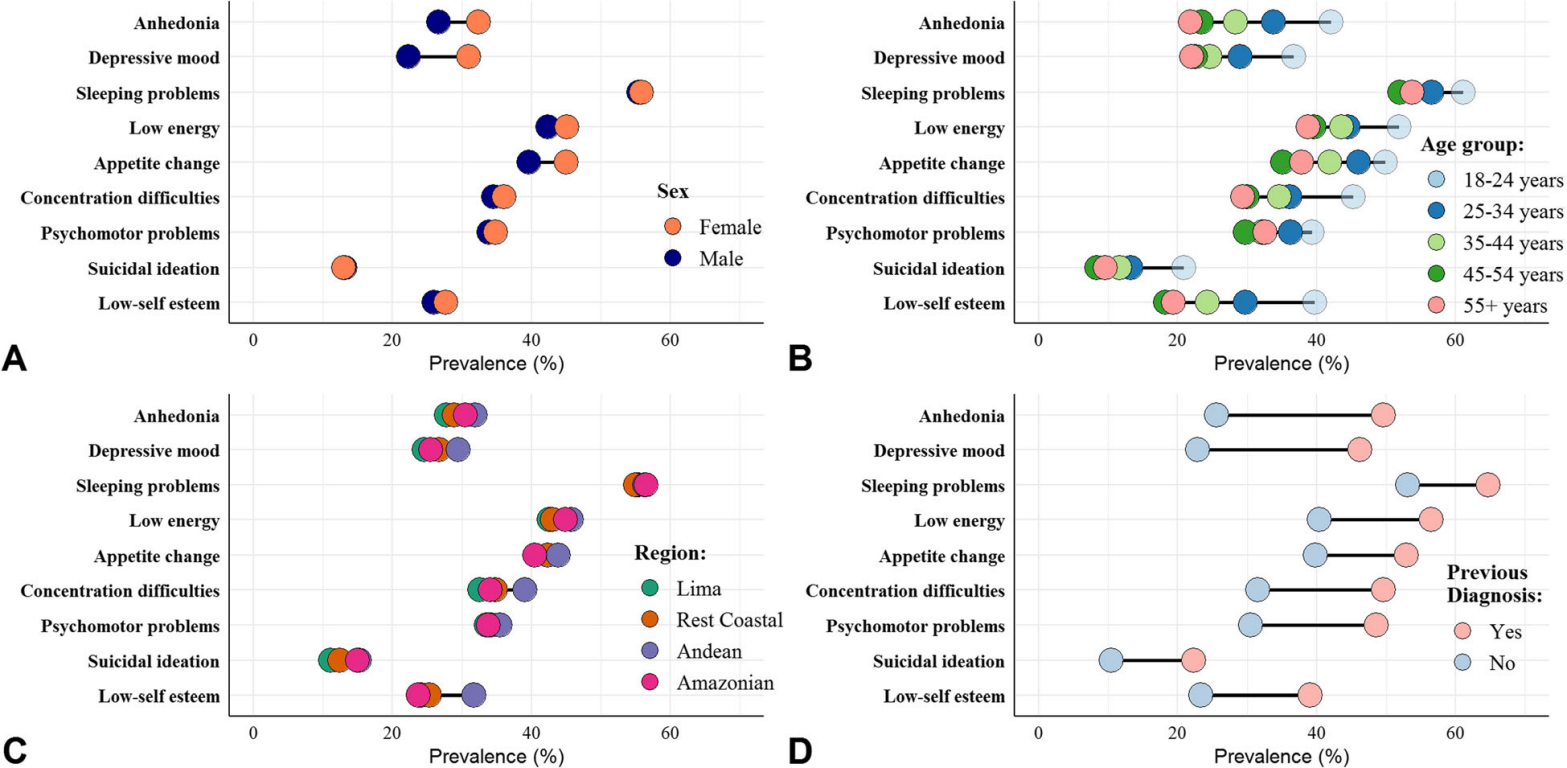
Population reporting mental health symptoms (PHQ-9) 7 days or more in the past 2 weeks by key strata. **a**: Sex, **b**: Age group, **c**: Geographical region, and, **d**: Prior mental health diagnosis

We found that more than half of the sample (62%) scored at least 2 points on the PHQ-2, making them eligible to complete all PHQ-9 items. Both items of the PHQ-2, related to anhedonia and depressive mood, affected nearly 30% of the sample most of the time (≥7 days) during the past 14 days. These symptoms were much more frequent among female and young participants. Figure 2 shows the differences in each item and PHQ-9 score by key strata.

**Fig. 3:**
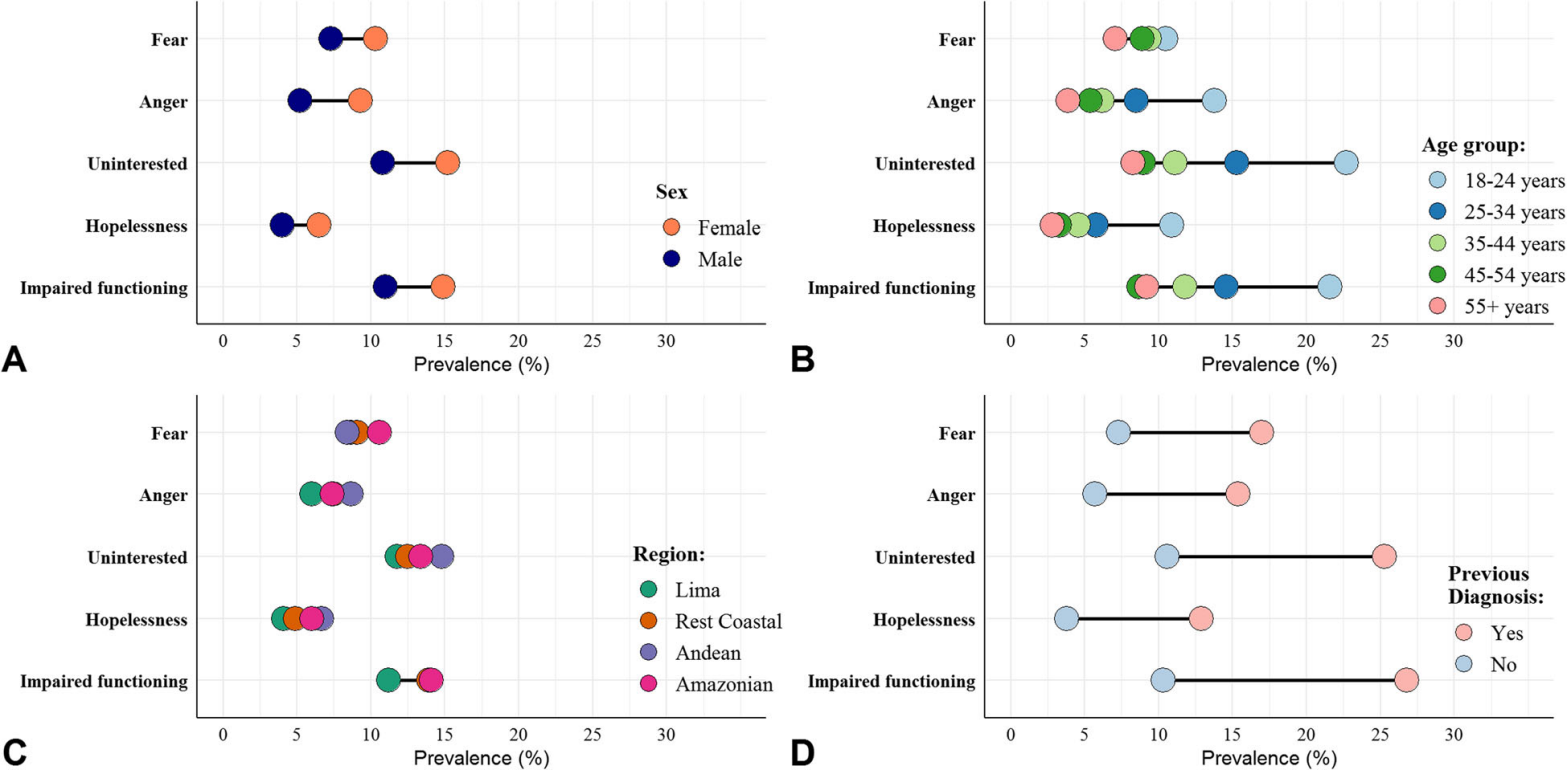
Population reporting mental health symptoms (WASSS) 7 days or more in the past 2 weeks by key strata. **a**: Sex, **b**: Age group, **c**: Geographical region, and, **d**: Prior mental health diagnosis

The 6-item WASSS showed additional psychosocial and functioning symptoms. Disparities by sex, age group, region, and previous mental health diagnosis were consistent with those in the PHQ-9. The absolute sex gap was the widest for feeling angry and out of control as well as for feeling uninterested and did not want to do anything at all most of the time during the past 2 weeks. Impaired functioning affected a fifth of the youngest participants, while hopelessness reached up to 10% for those with a previous mental health diagnosis. Figure 3 shows the differences in each WASSS item by key strata.

### Depressive symptoms and associated factors

The PHQ-2 score was highest among those with a previous mental health diagnosis and those who preferred not to answer that question. Similar disparity trends were found for most of the PHQ-9 items. Nearly 35% of respondents had a PHQ-9 score of 10 or more, linked to major depressive episodes. This was slightly higher in the Andes, followed by Metropolitan Lima.

The prevalence of depressive symptoms was 59% (95%CI 56.7, 61.4%) among participants who reported having a previous mental health diagnosis, which was similar compared to those who preferred not to respond to this question (60, 95%CI 54, 66.3%) and almost doubled the prevalence among those without a prior diagnosis (30, 95%CI 29.1, 30.9%). Table 2 shows the percentage and 95% confidence intervals of participants with a PHQ-9 score of 10 or more across covariate levels and by previous mental health diagnosis.

**Table 2:** Prevalence of depressive symptoms (PHQ-9 ≥ 10) by previous mental health diagnosis and key covariate levels, *n* = 57,446

	Previous Mental Health Diagnosis		
	Yes	No	NR
	***n*** = 9383	***n*** = 46,585	***n*** = 1282
	X% (95% CI)	X% (95% CI)	X% (95% CI)
**Sex**			
Male	53.6 (49.5, 57.7)	24.9 (23.7, 26.1)	60.8 (50.3, 70.5)
Female	62.2 (59.3, 64.9)	35.3 (34, 36.6)	59.9 (52, 67.4)
**Age group**			
18–24 years	73 (68.9, 76.7)	44.1 (42.4, 45.8)	74.7 (66.1, 81.7)
25–34 years	65.9 (62.1, 69.6)	35.5 (34, 37.1)	59.1 (48.4, 69)
35–55 years	58.3 (53.4, 63)	26.6 (24.8, 28.5)	48.7 (34.1, 63.5)
45–54 years	49.4 (43.4, 55.3)	23.4 (21.1, 25.8)	49 (29.7, 68.5)
55 years+	51.4 (45.1, 57.5)	21.7 (19.5, 24)	56.3 (36.9, 73.9)
**Region**			
Metropolitan Lima	58.2 (54.7, 61.7)	28.3 (26.9, 29.8)	65.1 (54.3, 74.5)
Resto Coast	60.1 (54.7, 65.3)	28 (26.3, 29.8)	62.8 (48.8, 75)
Andes	60.1 (55.9, 64.2)	33.5 (31.9, 35.2)	56.7 (46.8, 66.2)
Jungle	55.1 (45.3, 64.6)	29.2 (26.1, 32.5)	54.8 (34.5, 73.5)
**Education**			
Less than high school	61.4 (55, 67.3)	34.8 (32.4, 37.2)	63.9 (44.6, 79.5)
Completed high school	62.1 (59.1, 64.9)	31.2 (30.1, 32.3)	62.4 (55.2, 69)
Undergraduate or higher	49.4 (45.3, 53.5)	22.3 (21, 23.6)	46.7 (33.9, 60)
**Marital status**			
Single	67.5 (64.5, 70.4)	35.7 (34.4, 37)	67.3 (60, 73.9)
Married	47.3 (42.1, 52.6)	22.9 (21.1, 24.7)	53.9 (37.6, 69.5)
Living together	57.9 (52.7, 63)	28 (26.3, 29.8)	41.8 (28.8, 56.1)
Other	55.7 (48, 63.2)	32.3 (28.5, 36.3)	65.6 (38.8, 85.2)
**Household size**			
< 4	56.4 (52.7, 60.1)	28.8 (27.4, 30.2)	58.1 (48, 67.5)
≥ 4	61 (57.9, 64)	30.9 (29.8, 32.1)	62.1 (53.9, 69.6)
**Employment**			
Formal employment	44.4 (40.0, 49.0)	21.4 (19.9, 22.8).	45.7 (32.0, 60.2).
Informal employment	63.6 (57.1,69.6)	28.6 (26.4, 30.9)	39.3 (23.3, 58.0)
Unemployed before lockdown	64.6 (59.8, 69.2)	35.7 (33.6, 37.9)	68.5 (56.8, 78.2)
Recently unemployed	63.0 (59.2, 66.7)	34.2 (32.7,3 5.8)	68.8 (59.0, 77.0)
**Household income**			
Up to 930 PEN	63.8 (59.6, 67.9)	33.4 (31.8, 35.1)	56.2 (43.9, 67.9)
931–1860 PEN	57.9 (52.4, 63.1)	29.2 (27.2, 31.2)	60.7 (44.4, 75)
1861–2790 PEN	56.5 (48.8, 63.9)	26.5 (23.9, 29.3)	62.1 (39.3, 80.6)
2791–4650 PEN	45.9 (37.4, 54.7)	23.4 (20.6, 26.5)	61.8 (36, 82.3)
More than 4650 PEN	43.8 (35.8, 52.1)	19.2 (16.6, 22.1)	73.6 (50.6, 88.3)
Prefer not to respond	62.1 (57.5, 66.5)	31 (29.3, 32.8)	61.3 (51.4, 70.4)
**COVID-19 infection**			
No	59.1 (56.7, 61.5)	29.8 (29, 30.7)	60.1 (53.7, 66.2)
Yes	55.8 (32, 77.1)	43.9 (33.4, 54.9)	58.4 (11.3, 93.9)
**Comorbidities**			
No	57.8 (54.7, 60.8)	27.7 (26.7, 28.8)	58.9 (51.5, 65.9)
Yes	60.9 (56.9, 64.7)	34.3 (32.6, 36.1)	58.8 (46.8, 69.8)
**Total**	59 (56.7, 61.4)	30 (29.1, 30.9)	60.3 (54, 66.3)

X% (95% CI), values are presented as proportions and linearized 95% confidence intervals

Depressive symptoms trends by covariate level remained constant across these three groups, showing higher magnitudes in those with a prior diagnosis. There was a dose-response gradient observed by household income level, being higher in those with up to 930 PEN (274 USD) and the lowest with more than 4650 PEN (1368 USD), among those with or without a prior mental health diagnosis.

Regression models that included only sex and age revealed that females and young adults show a higher prevalence of depressive symptoms than their male or older counterparts. These results were consistent across mental health diagnosis strata. Sex-and-age adjusted regression revealed other associated factors: education level, marital status, household income, and presence of at least one chronic comorbidity. Participants with an undergraduate degree or higher had a lower prevalence than those with incomplete high school or less (PR: 0.76, 95%CI 0.69, 0.83, and PR: 0.86, 95%CI 0.76, 0.98; in the group with and without prior mental health diagnosis respectively).

Those reporting having a COVID-19 positive diagnosis did not score differently for depressive symptoms than non-cases among those with a previous mental health diagnosis. The dose-response relationship with income was observed despite the sex and age adjustment. The presence of any chronic disease comorbidity was associated with an increased risk for depressive symptoms reaching up to 43% more than in those without comorbidities. Risk and protective factors remain similar among those with and without prior mental health diagnosis, showing higher contrasts by region of residence.

An exception was the COVID-19 diagnosis history, which increased the risk for depressive symptoms by around 43% (PR: 1.43, 95%CI 1.34,1.53) among those without a prior mental health diagnosis. Table 3 shows the prevalence ratio and its corresponding 95% confidence intervals.

**Table 3:** Sex-and-aged adjusted prevalence ratios for risk factors of depressive symptoms (PHQ-9 ≥ 10) by previous mental health diagnosis, n = 57,446

	Previous Mental Health Diagnosis		
	Yes	No	NR
	***n*** = 9383	***n*** = 46,585	***n*** = 1282
	PR (95%CI)	PR (95%CI)	PR (95%CI)
**Region**			
Metropolitan Lima	Ref.	Ref.	Ref.
Resto Coast	1 (0.90,1.12)	0.90** (0.83,0.97)	0.97 (0.74,1.27)
Andes	0.96 (0.88,1.06)	1.04 (0.97,1.12)	0.81 (0.64,1.03)
Jungle	0.89 (0.73,1.07)	0.89 (0.79,1.01)	0.77 (0.52,1.14)
**Education**			
Less than high school	Ref.	Ref.	Ref.
Completed high school	1.02 (0.91,1.14)	0.93 (0.86,1.00)	0.96 (0.70,1.31)
Undergraduate or higher	0.86* (0.76,0.98)	0.76*** (0.69,0.83)	0.77 (0.51,1.16)
**Marital status**			
Single	Ref.	Ref.	Ref.
Married	0.78*** (0.68,0.90)	0.87** (0.79,0.96)	1.03 (0.68,1.57)
Living together	0.90* (0.81,0.99)	0.85*** (0.79,0.92)	0.7 (0.48,1.02)
Other	0.91 (0.77,1.07)	1.20** (1.05,1.37)	1.25 (0.78,2.02)
**Household size (≥4)**	1.06 (0.98,1.15)	1.03 (0.97,1.09)	1.05 (0.85,1.30)
**Household income**			
Up to 930 PEN	Ref.	Ref.	Ref.
931–1860 PEN	0.91 (0.82,1.02)	0.95 (0.87,1.03)	1.09 (0.78,1.54)
1861–2790 PEN	0.89 (0.76,1.03)	0.9 (0.80,1.01)	1.05 (0.70,1.56)
2791–4650 PEN	0.76** (0.62,0.93)	0.81** (0.71,0.93)	1.08 (0.70,1.69)
More than 4651 PEN	0.70*** (0.58,0.85)	0.68*** (0.58,0.79)	1.21 (0.84,1.74)
Prefer not to respond	0.97 (0.88,1.07)	0.94 (0.88,1.01)	1.09 (0.83,1.44)
**COVID-19 infection**	0.89 (0.58,1.36)	1.67*** (1.31,2.13)	0.88 (0.40,1.95)
**Comorbidities**	1.14** (1.05,1.24)	1.43*** (1.34,1.53)	1.09 (0.85,1.40)
**Employment**			
Formal employment	Ref.	Ref.	Ref.
Informal employment	1.37*** (1.19,1.58)	1.25*** (1.12,1.38)	0.81 (0.47,1.40)
Unemployed before lockdown	1.34*** (1.18,1.52)	1.40*** (1.271,1.53)	1.29 (0.88,1.87)
Recently unemployed	1.35*** (1.20,1.51)	1.42*** (1.31,1.53)	1.47* (1.04,2.07)

Values represent independent sex and age adjusted models for each covariate

*PR* prevalence ratio, *95%CI* 95% confidence intervals

*p-value< 0.05, ** *p*-value < 0.01, *** *p*-value < 0.001

### Secondary outcomes

Multivariate analysis for secondary outcomes revealed patterns similar to those depicted in the depressive symptoms results. A particular finding was the higher odds of outcomes in the Andes regions across mental health diagnosis strata. Lastly, those with higher household income showed consistently lower odds of impaired functioning than those at the minimum wage, exhibiting differences up to 50% lower odds than the poorest participants among those with a previous mental health diagnosis (OR: 0.50, 95%CI 0.43, 0.57).

### Sensitivity analyses

Results restricting those responses who completed the survey (*n* = 50,195) or including those with at least 75% of survey progress (*n* = 58,731) did not substantially change our main results. It is important to note that the latter group’s additional responses did not have data for household income, increasing the portion of missingness for this variable from 0.34 to 2.52%.

Sex-and-age adjusted regression estimates using the alternative cut-off point for depressive symptoms (PHQ-9 ≥ 14) showed similar patterns for key associated factors, including education, employment, and household income. Table 4 shows the regression estimates and corresponding 95% confidence intervals.

**Table 4:** Sex-and-aged adjusted prevalence ratios for risk factors of depressive symptoms (PHQ-9 ≥ 14) by previous mental health diagnosis, *n* = 57,446

	Previous Mental Health Diagnosis		
	Yes	No	NR
	***n*** = 9383	***n*** = 46,585	***n*** = 1282
	PR (95%CI)	PR (95%CI)	PR (95%CI)
**Region**			
Metropolitan Lima	Ref.	Ref.	Ref.
Resto Coast	1.19* (1.03,1.37)	0.91 (0.81,1.02)	1.2 (0.82,1.76)
Andes	1.07 (0.95,1.21)	1.09 (0.99,1.21)	0.88 (0.63,1.23)
Jungle	0.94 (0.73,1.21)	0.91 (0.77,1.07)	0.94 (0.57,1.55)
**Education**			
Less than high school	Ref.	Ref.	Ref.
Completed high school	1.02 (0.91,1.14)	0.80*** (0.72,0.88)	1.14 (0.70,1.87)
Undergraduate or higher	0.86* (0.76,0.98)	0.62*** (0.54,0.70)	0.92 (0.50,1.69)
**Marital status**			
Single	Ref.	Ref.	Ref.
Married	0.78*** (0.68,0.90)	0.85* (0.74,0.97)	1.18 (0.68,2.02)
Living together	0.90* (0.81,0.99)	0.79*** (0.71,0.87)	0.71 (0.44,1.15)
Other	0.91 (0.77,1.07)	1.16 (0.96,1.42)	1.18 (0.61,2.29)
**Household size (≥4)**	1.03 (0.92,1.15)	0.99 (0.91,1.08)	1.24 (0.92,1.68)
**Household income**			
Up to 930 PEN	Ref.	Ref.	Ref.
931–1860 PEN	0.91 (0.82,1.02)	0.89 (0.80,1.01)	1.02 (0.64,1.62)
1861–2790 PEN	0.89 (0.76,1.03)	0.81** (0.70,0.95)	1.13 (0.72,1.78)
2791–4650 PEN	0.76** (0.62,0.93)	0.72*** (0.59,0.87)	0.88 (0.47,1.63)
More than 4651 PEN	0.70*** (0.58,0.85)	0.60*** (0.48,0.75)	0.56 (0.29,1.09)
Prefer not to respond	0.97 (0.88,1.07)	0.91 (0.82,1.01)	0.95 (0.66,1.37)
**COVID-19 infection**	0.89 (0.58,1.36)	1.65** (1.14,2.39)	0.1 (0.01,1.01)
**Comorbidities**	1.14** (1.05,1.24)	1.49*** (1.36,1.63)	1.05 (0.73,1.50)
**Employment**			
Formal employment	Ref.	Ref.	Ref.
Informal employment	1.36** (1.11,1.66)	1.28** (1.11,1.48)	1.16 (0.57,2.36)
Unemployed before lockdown	1.55*** (1.31,1.84)	1.36*** (1.20,1.55)	1.59 (0.93,2.72)
Recently unemployed	1.50*** (1.28,1.75)	1.39*** (1.24,1.60)	1.64 (0.97,2.77)

Values represent independent sex and age adjusted models for each covariate

*PR* prevalence ratio, *95%CI* 95% confidence interval

*p-value< 0.05, ** *p*-value < 0.01, *** *p*-value < 0.001

## Discussion

We found a high mental health burden during the pandemic and lockdown in Peru. Three out of ten participants reported moderate to severe depressive symptoms in the prior 2 weeks. We found substantial disparities by key social variables favoring those with higher household income, education, and employment. Besides, we showed disparities of all psychosocial symptoms by sex, age group, and region. Stratified results by previous mental health diagnosis revealed a two-fold prevalence difference in depressive symptoms among those with a prior diagnosis compared to those never diagnosed. To our knowledge, this is the first study quantifying the mental health burden associated with the COVID-19 pandemic with a considerable sample size in Latin America.

Our findings reveal an overall prevalence of depressive symptoms five times higher than the one reported previously at a national level in 2018 (34.9% vs. 6.4%, respectively) [27]. Even if we limit our results to those without a prior diagnosed mental health condition, the magnitude of moderate to severe depressive symptoms is still nearly five times higher than the national prevalence. Risk factors such as being a woman, living in the Andes, and having comorbidities increased the probability of depressive symptoms.

Contrary to previous evidence in Peru [27, 28], we found that the younger population was more prone to having depressive symptoms. However, similar results have been found in large samples in the US [29] and the UK [30] during the pandemic, where young adults showed a higher prevalence of severe psychological distress compared to older groups [29]. Another possible explanation is that the pandemic and the measures implemented affect young people and adolescents differently due to their needs, socioeconomic condition, fear, and intolerance to uncertainty [31, 32] . Differences could also be attributed to young adults’ higher participation in web surveys, in this particular case participation of those with a prior mental health problem or experiencing psychological distress.

Population-based mental health evidence during the pandemic is limited. A recent study in China, which used a web-based survey and reached around 56,000 participants, found 28% of participants had depression, seven percentage points lower than our estimate [33]. There were no other comparable studies, especially in the Americas. Few studies with large sample sizes have been reported across the world. Results from Spain during the early stage of the pandemic [34] show that a third of Spaniards reported moderate to severe psychological impact, particularly women, the young, and the unemployed. Initial results from the COLLATE project suggest increased psychological distress among those with a mood disorder compared to those without reported mental disorders [35]. A social media-based survey in Taiwan [36] found that half of the participants and some 10% reported sleep disturbances and suicidal thoughts. Similar results were obtained in a small sample of Chinese [37] and Indian [38] adults, showing mild stress but high anxiety levels. Additional studies are being implemented to assess the impact of diverse physical distancing measures [39].

Our research provides a detailed characterization of mental health and psychological symptoms using a sample of around 60,000 respondents during the community transmission phase of the pandemic and lockdown in Peru. This massive participation was possible through a key partnership between PAHO and the Ministry of Health, in a time where the Peruvian Government was communicating COVID-19 information, interventions, plans, and restrictions in their official and social media accounts. Thus, the number of people visiting these sites was abundant, which was reflected in our online questionnaire’s participation rates. Moreover, we describe associated factors among participants by a previous mental health condition, providing relevant information for mental health action plans in the COVID-19 pandemic context. For instance, this study’s preliminary results were shared with the Ministry of Health in Peru to plan its mental health response [40].

The study has some limitations that are important to consider for the correct interpretations of our findings. First, we did not include other mental health issues such as psychological distress or anxiety in this study. Unlike depression, there were not previous studies on anxiety or distress at the national level to be used as a pre-COVID reference. We believe it is very likely that these problems are also present during this pandemic and lockdown; however, quantifying these problems was beyond the scope of our study.

We used an opt-in online survey using social media as the distribution channel to collect information from respondents who had internet access and consented to participate. Internet connectivity in Peru has substantially increased in the past decade, representing 81 and 46.8% among 17–24 and 25+ years old citizens, respectively [41]. Urban areas and the Coastal region have more increased connectivity than their rural, Andean, or Amazonian counterparts. Thus, regional or national results represent those with access to internet connectivity, usually excluding the poorest and most vulnerable.

Older adults are the smallest group of social media users; thus, we expected to find a low number of respondents of this age group. Besides adjusting for lower participation in the older population using raking, we encouraged all respondents who stated there is an older adult in the household to complete the survey for themselves. We did not have information about the rurality of the residence. Rural areas are more prevalent in the Andean and Amazonian regions; however, sex-age adjusted models did not found substantial differences by geographical region. We addressed these limitations using raking weights, which optimally balanced the data to known national marginal proportions of age, sex, education, and region of residence from the latest census official data in 2017.

Lastly, due to the survey’s opt-in nature, selection bias such as volunteer bias is possible. Participants who are experiencing psychological distress or have an existing mental health condition are more likely to participate and complete the questionnaire. We addressed this issue by providing stratified results by previous mental health conditions, which uncovered a depressive symptoms prevalence of 30%, a 5-fold increase with respect to national levels reported in 2019.

An increased burden of depressive symptoms and psychosocial reactions emerged during the current pandemic and national lockdown in Peru. The mental health burden is disproportionately affecting participants with lower income and education as well as those unemployed. Peru has proven to be a dynamic country in terms of mental health reform and transitioning from a hospital-centered mental health model towards a community-based model whereby community networks are being established. As the Government is relaxing the social distancing measures and expanding its economic reactivation to phase four, with most businesses open with some restrictions, it is crucial to use local evidence to adjust public health policies and services in mental health to the renewed needs of the Peruvian people.

## Supplementary Information

**Additional file 1: Supplementary File 1**. English version of the main questionnaire.

**Additional file 2: Supplementary File 2**. Spanish version of the main questionnaire.

**Additional file 3: Supplementary File 3**. PHQ-9 and WASSS items.

**Additional file 4: Supplementary File 4**. Variables values in the analytical sample and Peruvian Census 2017* used for raking.

## Data Availability

The datasets generated and/or analysed during the current study are not publicly available but are available from the corresponding author on reasonable request.

## References

[1] Adhanom Ghebreyesus T (2020). Addressing mental health needs: an integral part of COVID-19 response. World Psychiatry.

[2] The Lancet P (2020). Mental health and COVID-19: change the conversation. Lancet Psychiatry.

[3] IASC Reference Group on MHPSS in Emergency Settings: Interim Briefing Note Addressing Mental Health and Psychosocial Aspects of COVID-19 Outbreak. Version 1.5. In.: Inter Agency Standing Committee (IASC). Lancet Psychiatry; 2020. https://www.thelancet.com/journals/lanpsy/article/PIIS2215-0366(20)30194-2/fulltext#articleInformation.

[4] Rogers JP, Chesney E, Oliver D, Pollak TA, McGuire P, Fusar-Poli P, Zandi MS, Lewis G, David AS. Psychiatric and neuropsychiatric presentations associated with severe coronavirus infections: a systematic review and meta-analysis with comparison to the COVID-19 pandemic. Lancet Psychiatry. 2020;7(7):611–27. 10.1016/S2215-0366(20)30203-0. Epub 2020 May 18. 10.1016/S2215-0366(20)30203-0 PMC7234781 32437679

[5] Paterson RW, Brown RL, Benjamin L, et al. The emerging spectrum of COVID-19 neurology: clinical, radiological and laboratory findings. Brain 2020;143(10):3104–20. 10.1093/brain/awaa240 PMC7454352 32637987

[6] de Girolamo G, Cerveri G, Clerici M, et al. Mental Health in the Coronavirus Disease 2019 Emergency—The Italian Response. JAMA Psychiatry. 2020;77(9):974–6. 10.1001/jamapsychiatry.2020.1276. 10.1001/jamapsychiatry.2020.1276 32352480

[7] Galea S, Merchant RM, Lurie N. The Mental Health Consequences of COVID-19 and Physical Distancing: The Need for Prevention and Early Intervention. JAMA Intern Med. 2020;180(6):817–8. 10.1001/jamainternmed.2020.1562 10.1001/jamainternmed.2020.1562 32275292

[8] World Health Organization. WHO Director-General's opening remarks at the media briefing on COVID-19. 2020. https://www.who.int/dg/speeches/detail/who-director-general-s-opening-remarks-at-the-media-briefing-on-covid-19---20-july-2020. Accessed 23 July 2020.

[9] Cumulative COVID-19 cases. https://ais.paho.org/phip/viz/COVID19Table.asp. Accessed 16 Dec 2020.

[10] Boadle A. WHO says the Americas are new COVID-19 epicenter as deaths surge in Latin America. Reuters; 2020. https://www.nytimes.com/reuters/2020/05/26/world/americas/26reuters-health-coronavirus-latam.html. Accessed 26 May 2020.

[11] Antiporta DA, Bruni A. Emerging mental health challenges, strategies, and opportunities in the context of the COVID-19 pandemic: perspectives from South American decision-makers. Rev Panam Salud Publica. 2020;44:e154. 10.26633/RPSP.2020.154. 10.26633/RPSP.2020.154 PMC7679046 33245299

[12] Rossi R, Socci V, Pacitti F, Di Lorenzo G, Di Marco A, Siracusano A, Rossi A (2020). Mental health outcomes among frontline and second-line health care workers during the coronavirus disease 2019 (COVID-19) pandemic in Italy. JAMA Netw Open.

[13] Huang Y, Zhao N (2020). Chinese mental health burden during the COVID-19 pandemic. Asian J Psychiatr.

[14] Xie X, Xue Q, Zhou Y, et al. Mental Health Status Among Children in Home Confinement During the Coronavirus Disease 2019 Outbreak in Hubei Province, China. JAMA Pediatr. 2020;174(9):898–900. 10.1001/jamapediatrics.2020.1619. 10.1001/jamapediatrics.2020.1619 PMC7182958 32329784

[15] Amsalem D, Dixon LB, Neria Y. The Coronavirus Disease 2019 (COVID-19) Outbreak and Mental Health: Current Risks and Recommended Actions. JAMA Psychiatry. 2021;78(1):9–10. 10.1001/jamapsychiatry.2020.1730 10.1001/jamapsychiatry.2020.1730 32579160

[16] Gasser U, Ienca M, Scheibner J, Sleigh J, Vayena E. Digital tools against COVID-19: taxonomy, ethical challenges, and navigation aid. Lancet Digit Health. 2020;2(8):e425–e434. 10.1016/S2589-7500(20)30137-0. Epub 2020 Jun 29. 10.1016/S2589-7500(20)30137-0 PMC7324107 32835200

[17] Whitelaw S, Mamas MA, Topol E, Van Spall HGC. Applications of digital technology in COVID-19 pandemic planning and response. Lancet Digit Health. 2020;2(8):e435–e440. 10.1016/S2589-7500(20)30142-4. Epub 2020 Jun 29. 10.1016/S2589-7500(20)30142-4 PMC7324092 32835201

[18] World Health Organization & United Nations High Commissioner for Refugees (2012). Assessing Mental Health and Psychosocial Needs and Resources: Toolkit for Humanitarian Settings.

[19] Calderón M, Gálvez-Buccollini JA, Cueva G, Ordoñez C, Bromley C, Fiestas F (2012). Validation of the Peruvian version of the PHQ-9 for diagnosing depression. Rev Peru Med Exp Salud Publica.

[20] Villarreal-Zegarra D, Copez-Lonzoy A, Bernabé-Ortiz A, Melendez-Torres GJ, Bazo-Alvarez JC (2019). Valid group comparisons can be made with the patient health questionnaire (PHQ-9): a measurement invariance study across groups by demographic characteristics. Plos One.

[21] Arroll B, Goodyear-Smith F, Crengle S, Gunn J, Kerse N, Fishman T, Falloon K, Hatcher S (2010). Validation of PHQ-2 and PHQ-9 to screen for major depression in the primary care population. Ann Fam Med.

[22] Levis B, Sun Y, He C, Wu Y, Krishnan A, Bhandari PM, Neupane D, Imran M, Brehaut E, Negeri Z (2020). Accuracy of the PHQ-2 alone and in combination with the PHQ-9 for screening to detect major depression: systematic review and meta-analysis. JAMA.

[23] Poole DN, Liao S, Larson E, Hedt-Gauthier B, Raymond NA, Barnighausen T, Smith Fawzi MC (2020). Sequential screening for depression in humanitarian emergencies: a validation study of the patient health questionnaire among Syrian refugees. Ann General Psychiatry.

[24] Levis B, Benedetti A, Thombs BD (2019). Accuracy of patient health Questionnaire-9 (PHQ-9) for screening to detect major depression: individual participant data meta-analysis. BMJ.

[25] Instituto Nacional de Estadística e Informática (2018). Censos nacionales 2017: XII de población, VII de vivienda y II de comunidades indígenas. Sistema de consulta de datos (REDATAM).

[26] Levis B, Benedetti A, Ioannidis JPA, Sun Y, Negeri Z, He C, Wu Y, Krishnan A, Bhandari PM, Neupane D (2020). Patient health Questionnaire-9 scores do not accurately estimate depression prevalence: individual participant data meta-analysis. J Clin Epidemiol.

[27] Hernandez-Vasquez A, Vargas-Fernandez R, Bendezu-Quispe G, Grendas LN (2020). Depression in the Peruvian population and its associated factors: analysis of a national health survey. J Affect Disord.

[28] Martina M, Ara MA, Gutiérrez C, Nolberto V, Piscoya J (2017). Depresión y factores asociados en la población peruana adulta mayor según la ENDES 2014-2015. Anales de la Facultad de Medicina.

[29] McGinty EE, Presskreischer R, Han H, Barry CL. Psychological Distress and Loneliness Reported by US Adults in 2018 and April 2020. JAMA. 2020;324(1):93–4. 10.1001/jama.2020.9740. 10.1001/jama.2020.9740 PMC7270868 32492088

[30] O'Connor RC, Wetherall K, Cleare S, McClelland H, Melson AJ, Niedzwiedz CL, O'Carroll RE, O'Connor DB, Platt S, Scowcroft E, at al. Mental health and well-being during the COVID-19 pandemic: longitudinal analyses of adults in the UK COVID-19 Mental Health & Wellbeing study. Br J Psychiatry. 2020;21:1–8. 10.1192/bjp.2020.212. Epub ahead of print. 10.1192/bjp.2020.212 PMC7684009 33081860

[31] Glowacz F, Schmits E (2020). Psychological distress during the COVID-19 lockdown: the young adults most at risk. Psychiatry Res.

[32] Singh S, Roy D, Sinha K, Parveen S, Sharma G, Joshi G (2020). Impact of COVID-19 and lockdown on mental health of children and adolescents: a narrative review with recommendations. Psychiatry Res.

[33] Shi L, Lu Z-A, Que J-Y, Huang X-L, Liu L, Ran M-S, Gong Y-M, Yuan K, Yan W, Sun Y-K (2020). Prevalence of and risk factors associated with mental health symptoms among the general population in China during the coronavirus disease 2019 pandemic. JAMA Netw Open.

[34] Rodríguez-Rey R, Garrido-Hernansaiz H, Collado S (2020). Psychological impact and associated factors during the initial stage of the coronavirus (COVID-19) pandemic among the general population in Spain. Front Psychol.

[35] Van Rheenen TE, Meyer D, Neill E, Phillipou A, Tan EJ, Toh WL, Rossell SL (2020). Mental health status of individuals with a mood-disorder during the COVID-19 pandemic in Australia: initial results from the COLLATE project. J Affect Disord.

[36] Li DJ, Ko NY, Chen YL, Wang PW, Chang YP, Yen CF, Lu WH. COVID-19-Related Factors Associated with Sleep Disturbance and Suicidal Thoughts among the Taiwanese Public: A Facebook Survey. Int J Environ Res Public Health. 2020;17(12):4479. 10.3390/ijerph17124479. 10.3390/ijerph17124479 PMC7345275 32580433

[37] Zhang Y, Ma ZF (2020). Impact of the COVID-19 pandemic on mental health and quality of life among local residents in Liaoning Province, China: a cross-sectional study. Int J Environ Res Public Health.

[38] Roy D, Tripathy S, Kar SK, Sharma N, Verma SK, Kaushal V (2020). Study of knowledge, attitude, anxiety & perceived mental healthcare need in Indian population during COVID-19 pandemic. Asian J Psychiatr.

[39] Giallonardo V, Sampogna G, Del Vecchio V, Luciano M, Albert U, Carmassi C, Carra G, Cirulli F, Dell'Osso B, Nanni MG (2020). The impact of quarantine and physical distancing following COVID-19 on mental health: study protocol of a multicentric Italian population trial. Front Psychiatry.

[40] Resolución Ministerial N° 363-2020-MINSA. Plan de Salud Mental en el contexto Covid-19 - Perú, 2020 - 2021. Lima: 2020.

[41] Instituto Nacional de Estadística e Informática (2019). Encuesta Nacional de Hogares 2018.

