# Supplementary material for: Depressive symptoms among Peruvian adult residents amidst a National Lockdown during the COVID-19 pandemic
Source: BMC Psychiatry. 2021 Feb 18;21:111. doi: 10.1186/s12888-021-03107-3 (PMC7890781; doi:10.1186/s12888-021-03107-3)
Supplement: Supplementary file 1 — Additional file 1: Supplementary File 1. English version of the main questionnaire. [file 12888_2021_3107_MOESM1_ESM.pdf]

Questionnaire

Have you previously participated in this survey?
Yes
No

The reason you're filling out the survey is:
To send someone else's information
To correct my previous answers
To see the questions again
I'd rather not answer
Other (specify)

Sex:
Man
Woman
I'd rather not answer

What's your age?
18-24 years old
25-34 years old
35-44 years old
45-54 years old
55-64 years old
65-74 years old
75 years or older

What's your marital status?
Single
Married
Widower
Conviviant
Other

Are you complying with quarantine at home?
Yes
No
I'd rather not answer

As for where you slept yesterday, where are you located?
Department
Province
District

How many people, including you, slept in the same place where you stayed yesterday?
(Example: if you live it would only be 1)

How many people 60 years of age or older slept in the same place where you stayed yesterday, not including you?
(Example: if you are the only person in your 60s, put none)
Do you live in the same residence where you lived 6 months ago?

Yes
No
I'd rather not answer

Fill in the details of your previous residence:
Department
Province
District

Has your occupation been considered essential during quarantine?
No, my occupation has not been regarded as essential
Health services
Communications
Finance
Police and Armed Forces
Agricultural production or sale
Pharmaceutical production or sale
Other (specify)
I'd rather not answer

Has any household member been considered an essential worker?
Check all the options that apply.
No, no one in my household has been considered an essential worker
Health services
Communications
Finance
Police and Armed Forces
Agricultural production or sale
Pharmaceutical production or sale
Other (specify)
I'd rather not answer

What is the highest degree of education you have obtained or approved?
Incomplete primary
Full primary
Incomplete high school
Full high school
Incomplete Technique or Superior
Complete Technique or Superior
No formal instruction
Other (specify):
I'd rather not answer

The following questions discuss your level of well-being and mental health.

In the last two weeks, what has been the biggest concern you've ever had?

Have you sought help or support from mental health services in the last two weeks (i.e., between $-date://OtherDate/PT/-2%20week and yesterday)?
Yes
No
I'd rather not answer

Have you been diagnosed with any mental health problems in the past?
Yes
No
I'd rather not answer

What was your diagnosis?

When was he diagnosed?

Have you had any contact with mental health services in the last two weeks (i.e. between $date://OtherDate/PT/-2%20week and yesterday)?
Yes
No
I'd rather not answer

Point out the means by which you have contacted mental health services:
Check all appropriate options.
Phone calls
Text messages (Whatsapp, etc.)
Video Conferencing (Internet)
Other

Have you had any problems or challenges in accessing your mental health services in the last two weeks (i.e. between $-date://OtherDate/PT/-2%20week- and yesterday)?
Check all applicable options.
Dating
Recipes / Medicines
Non-pharmacological therapies (counseling services, psychology, etc.)
Periodic tracking (virtual or physical)
Other (specify):
I haven't had any problems.

What has been the biggest problem you've had with regard to mental health services in the last two weeks (between $-date://OtherDate/PT/-2%20week- and yesterday)?

How often over the past two weeks, i.e. between $date://OtherDate/PT/-2%20week and yesterday, you've felt discomfort or problems like:

	Not at all	Several days (1 to 6 days)	Most days (7 to 11 days)	Almost every day (12 to + days)	I'd rather not answer

Little desire or interest in doing things?	
Feeling discouraged, depressed, sad, or hopeless?	

How often over the past two weeks, i.e. between $date://OtherDate/PT/-2%20week and yesterday, you've felt discomfort or problems like:

	Not at all	Several days (1 to 6 days)	Most days (7 to 11 days)	Almost every day (12 to + days)	I'd rather not answer
Problems sleeping or staying asleep, or sleeping too much?					
Feeling tired or having little energy for no reason to justify it?					
Little appetite or overeating?					
Difficulty paying attention or concentrating on the things you do?					
Move slower or talk slower than normal or feel more restless or uneasy than normal?					
Thoughts that it would be better to be dead or that you wanted to hurt yourself in some way looking to die?					
Feel unwell about yourself or feel that you are a failed or that you have failed yourself or your family?					

These inconveniences or problems, how much have you had difficulty doing your job, your activities at home or getting along with other people?
A lot
Little
Nothing
I'd rather not answer

The following questions discuss your health status.
Have you ever been diagnosed with COVID-19?
Yes
No
I'd rather not answer

Have you ever had a COVID-19 diagnostic test? *(Molecular or rapid testing)*
Yes
No
I'd rather not answer

Have you been in contact with anyone with COVID-19?
Yes
No
I do not know

In the last 24 hours, have you experienced any of the following symptoms?
Check all the alternatives that apply.
Fever
Cough
Sore throat
Difficulty breathing
Muscle pain
Lack of taste or smell
Headache
Fatigue
Chest pain
Nausea
Eye pain
Nasal discharge
No symptoms

Do you currently suffer from any of these diseases?
Check all that apply.
Obesity
Arterial Hypertension
Another heart disease
Diabetes
Cancer
Immune Disease
No disease

In the last two weeks, i.e. between $date://OtherDate/PT/-2%20week and yesterday, has your consumption increased by:

	Yes	No	I'd rather not answer
Alcohol			
Tobacco and/or cigarettes			

Other substances	

How often over the last two weeks, i.e. between $date://OtherDate/PT/-2%20week and yesterday, you

	Always	Almost always	Sometimes	Rarely	Never	I'd rather not answer
Did he feel so scared that nothing could calm him down?						
Were you so angry that you felt out of control?						
Did he feel so disinterested in the things he used to like that he didn't feel like doing anything anymore?						
Did you feel so hopeless that you didn't want to go on living?						
Did you feel so upset about the emergency or other event in your life that you tried to avoid places, people, conversations or activities that might remind you of such an event?						
felt unable to perform essential activities of daily life because of these feelings?						

Please complete the following information about ALL people 60 years of age or older who live with you.

During the last two weeks this person...

	Age (years)	Sex	... felt so distressed/upset/annoyed that she was unable to carry out basic activities of daily life because of any of these feelings?	... are you acting strangely or having seizures or seizures?
#1		M ... F	If ... I'd rather not answer	If ... I don't know.
#2		M ... F	If ... I'd rather not answer	If ... I don't know.
#3		M ... F	If ... I'd rather not answer	If ... I don't know.
#4		M ... F	If ... I'd rather not answer	If ... I don't know.
#5		M ... F	If ... I'd rather not answer	If ... I don't know.

This last part researches socioeconomic information about you or your current home:
Are you the head of the household?
Yes
No
What is your link to the head of the household?
Father/Mother
Husband
Child

Brother
Other (specify):
I'd rather not answer

What was your employment situation before quarantine (before March 16, 2020)?
Formal worker
Informal work
I didn't have a job.

Did your work situation change during quarantine?
Yes
No

What is your employment situation during quarantine (after March 16, 2020)?
Formal work
Informal work
I don't have a job.

Has your household received any financial or other assistance from the government?
Check all applicable options.
Bonus "I stay home"
Independent bonus
Universal Family Bonus
Bonus (essential professional)
Food basket
Pension 65
Together
Popular Dining Room
Another kind of help
I'd rather not answer
I haven't received any help.

Currently, what is your household's total monthly income? Enter the most appropriate category.
Total monthly income is the sum of the income of all household members.
Up to 930 PEN
Between 931 and 1860 PEN
Between 1861 and 2790 PEN
Between 2791 and 4650 PEN
Between 4651 and 6510 PEN
Between 6511 and 9300 PEN
More than 9300 PEN
I'd rather not answer

Please put the figure corresponding to the total monthly income of your household, in PEN.
(example, 3050 for three thousand fifty PEN)
