# Supplementary material for: Depressive symptoms among Peruvian adult residents amidst a National Lockdown during the COVID-19 pandemic
Source: BMC Psychiatry. 2021 Feb 18;21:111. doi: 10.1186/s12888-021-03107-3 (PMC7890781; doi:10.1186/s12888-021-03107-3)
Supplement: Supplementary file 2 — Additional file 2: Supplementary File 2. Spanish version of the main questionnaire. [file 12888_2021_3107_MOESM2_ESM.pdf]

Cuestionario

¿Ha participado con anterioridad en esta encuesta?
Si
No

La razón por la que está llenando la encuesta es:
Para enviar información de otra persona
Para corregir mis respuestas anteriores
Para ver de nuevo las preguntas
Prefiero no responder
Otro (especifique)

Sexo:
Hombre
Mujer
Prefiero no responder

¿Cuál es su edad?
18-24 años
25-34 años
35-44 años
45-54 años
55-64 años
65-74 años
75 años o más

¿Cuál es su estado civil?
Soltero(a)
Casado(a)
Viudo(a)
Conviviente
Otro

¿Se encuentra cumpliendo la cuarentena en su hogar?
Si
No
Prefiero no contestar

Respecto al lugar en el que durmió ayer, ¿dónde se encuentra ubicado?
Departamento
Provincia
Distrito

¿Cuántas personas, incluyéndolo a usted, durmieron en el mismo lugar dónde usted se quedó ayer?
(Ejemplo: si vive solo sería 1)

¿Cuántas personas de 60 o más años de edad durmieron en el mismo lugar dónde usted se quedó ayer, sin incluirlo a usted?
(Ejemplo: si usted es la única persona de 60 años, poner ninguna)

¿Vive usted en la misma residencia donde vivía hace 6 meses?
Si
No
Prefiero no contestar

Complete los datos de su residencia anterior:
Departamento
Provincia
Distrito

¿Su ocupación ha sido considerada como esencial durante la cuarentena?
No, mi ocupación no ha sido considerada como esencial
Servicios de salud
Comunicaciones
Finanzas
Policia y Fuerzas Armadas
Producción o venta agropecuaria
Producción o venta farmaceútica
Otro (especifique)
Prefiero no responder

¿Algún miembro del hogar ha sido considerado como trabajador esencial?
Marque todas las opciones que apliquen.
No, nadie en mi hogar ha sido considerado como trabajador esencial
Servicios de salud
Comunicaciones
Finanzas
Policia y Fuerzas Armadas
Producción o venta agropecuaria
Producción o venta farmaceútica
Otro (especifique)
Prefiero no responder

¿Cúal es el grado de educación más alto que ha obtenido o aprobado?
Primaria incompleta
Primaria completa
Secundaria incompleta
Secundaria completa
Técnica o Superior incompleta
Técnica o Superior completa
Sin instrucción formal
Otro (especifique):
Prefiero no contestar

Las siguientes preguntas indagan sobre su nivel de bienestar y salud mental.

En las últimas dos semanas, ¿cuál ha sido la mayor preocupación que ha tenido?

¿Ha buscado ayuda o soporte de los servicios de salud mental en las últimas dos semanas (es decir, entre el ${date://OtherDate/PT/-2%20week} y el día de ayer)?
Si
No
Prefiero no responder

¿Ha sido diagnosticado con algún problema de salud mental en el pasado?
Si
No
Prefiero no contestar

¿Cuál fue su diagnóstico?

¿Cuando fue diagnosticado?

¿Ha tenido algún contacto con los servicios de salud mental en las últimas dos semanas (es decir, entre el ${date://OtherDate/PT/-2%20week} y el día de ayer)?
Si
No
Prefiero no contestar

Señale los medios por los cuáles se ha contactado con los servicios de salud mental:
Marque todas las opciones que correspondan.
Llamadas teléfonicas
Mensajes de texto (Whatsapp, etc.)
Videoconferencia (Internet)
Otro

¿Ha tenido algún problema o desafío en acceder a sus servicios de salud mental en las últimas dos semanas (es decir, entre el ${date://OtherDate/PT/-2%20week} y el día de ayer)?
Marque todas las opciones que correspondan.
Citas
Recetas / Medicamentos
Terapias no farmacológicas (servicios de consejería, psicología, etc.)
Seguimiento periódico (virtual o físico)
Otro (especifique):
No he tenido ningún problema

¿Cuál ha sido el mayor problema que ha tenido con respecto a los servicios de salud mental en las últimas dos semanas (entre el ${date://OtherDate/PT/-2%20week} y el día de ayer)?

¿Con qué frecuencia durante las últimas dos semanas, es decir, entre el ${date://OtherDate/PT/-2%20week} y el día de ayer, usted ha sentido molestias o problemas como:

	Para nada	Varios días (1 a 6 días)	La mayoría de días (7 a 11 días)	Casi todos los días (12 a + días)	Prefiero no responder
Pocas ganas o interés en hacer las cosas?					
Sentirse desanimada(o), deprimida(o), triste o sin esperanza?					

¿Con qué frecuencia durante las últimas dos semanas, es decir, entre el ${date://OtherDate/PT/-2%20week} y el día de ayer, usted ha sentido molestias o problemas como:

	Para nada	Varios días (1 a 6 días)	La mayoría de días (7 a 11 días)	Casi todos los días (12 a + días)	Prefiero no responder
Problemas para dormir o mantenerse dormida(o), o en dormir demasiado?					
Sentirse cansada(o) o tener poca energía sin motivo que lo justifique?					
Poco apetito o comer en exceso?					
Dificultad para poner atención o concentrarse en las cosas que hace?					
Moverse más lento o hablar más lento de lo normal o sentirse más inquieta(o) o intranquila(o) de lo normal?					
Pensamientos de que sería mejor estar muerta(o) o que quisiera hacerse daño de alguna forma buscando morir?					
Sentirse mal acerca de si misma(o) o sentir que es una(un) fracasada(o) o que se ha fallado a sí misma(o) o a su familia?					

Estas molestias o problemas, cuánto le han dificultado para realizar su trabajo, sus actividades en la casa o llevarse bien con otras personas?
Mucho
Poco
Nada
Prefiero no responder

Las siguientes preguntas indagan sobre su estado de salud.
¿Ha sido alguna vez diagnosticado con COVID-19?
Si
No
Prefiero no responder

¿Se le ha hecho alguna vez una prueba de diagnóstico de COVID-19? ***(Prueba molecular o rápida)***
Si
No
Prefiero no responder

¿Ha estado en contacto con alguna persona con COVID-19?
Si
No
No sé

¿En las últimas 24 horas, ha sufrido alguno de los siguientes síntomas?
Marque todas las alternativas que apliquen.
Fiebre
Tos
Dolor de garganta
Dificultad para respirar
Dolor muscular
Falta de gusto u olfato
Dolor de cabeza
Fatiga
Dolor de pecho
Nausea
Dolor de ojo
Secreción nasal
Ningún síntoma

¿Sufre actualmente de alguna de estas enfermedades?
Marque todas las que apliquen.
Obesidad
Hipertensión Arterial
Otra enfermedad cardiaca
Diabetes
Cáncer
Enfermedad Immune
Ninguna enfermedad

En las últimas dos semanas, es decir, entre el ${date://OtherDate/PT/-2%20week} y el día de ayer, ¿ha AUMENTADO su consumo de:

	Si	No	Prefiero no responder
Alcohol			
Tabaco y/o cigarrillos			
Otras sustancias			

¿Con qué frecuencia durante las últimas dos semanas, es decir, entre el ${date://OtherDate/PT/-2%20week} y el día de ayer, usted

	Siempre	Casi siempre	Algunas veces	Pocas veces	Nunca	Prefiero no responder
Se sintió tan asustado que nada lo podía calmar?						
Estuvo tan enfadado que se sintió fuera de control?						
Se sintió tan desinteresado por las cosas que acostumbraban a gustarle que ya no le apetecía hacer absolutamente nada?						
Se sintió tan desesperanzado que no quería seguir viviendo?						
Se sintió tan molesto por la emergencia u otro suceso de su vida, que intentó evitar lugares, personas, conversaciones o actividades que le pudieran recordar dicho evento?						
se sintió incapaz de realizar actividades esenciales de la vida						

diaria por culpa de estos sentimientos?

Complete la siguiente información sobre TODAS las personas de 60 o más años de edad que habitan con usted.

¿Durante las últimas dos semanas esta persona...

	Edad (años)	Sexo	...se sintió tan angustiada/trastornada/molesta que fue incapaz de llevar a cabo actividades básicas de la vida diaria debido a alguno de estos sentimientos?	... está actuando de manera extraña o teniendo ataques epilépticos o convulsiones?
#1		▼ M ... F	▼ Si ... Prefiero no responder	▼ Si ... No sé
#2		▼ M ... F	▼ Si ... Prefiero no responder	▼ Si ... No sé
#3		▼ M ... F	▼ Si ... Prefiero no responder	▼ Si ... No sé
#4		▼ M ... F	▼ Si ... Prefiero no responder	▼ Si ... No sé
#5		▼ M ... F	▼ Si ... Prefiero no responder	▼ Si ... No sé

Esta última parte indaga sobre información socioeconómica de usted o su hogar actual:
¿Es usted el jefe del hogar?

Si
No

¿Cuál es su vínculo con el jefe del hogar?
Padre/Madre
Esposo(a)
Hijo(a)
Hermano(a)
Otro (especifique):
Prefiero no responder

¿Cuál era su situación laboral antes de la cuarentena (antes del 16 marzo del 2020)?
Trabajador formal
Trabajo informal
No tenía trabajo

¿Cambió su situación laboral durante la cuarentena?
Si
No

¿Cuál es su situación laboral durante la cuarentena (después de marzo 16 del 2020)?
Trabajo formal
Trabajo informal
No tengo trabajo

¿Tu hogar ha recibido alguna ayuda económica o de otro tipo de parte del Gobierno?
Marque todas las opciones que correspondan.
Bono "Yo me quedo en casa"
Bono independiente
Bono Familiar Universal
Bono (profesional esencial)
Canasta de alimentos
Pensión 65
Juntos
Comedor Popular
Otro tipo de ayuda
Prefiero no responder
No he recibido ninguna ayuda

Actualmente, ¿cuál es el ingreso mensual total de su hogar? Indique la categoría más adecuada.
El ingreso mensual total es la suma de los ingresos de todos los miembros del hogar.
Hasta 930 soles
Entre 931 a 1860 soles
Entre 1861 a 2790 soles
Entre 2791 a 4650 soles
Entre 4651 a 6510 soles
Entre 6511 a 9300 soles
Más de 9300 soles
Prefiero no responder

Por favor, ponga la cifra correspondiente al ingreso mensual total de su hogar, en soles.
(ejemplo, 3050 para tres mil cincuenta soles)
