# Supplementary material for: Depressive symptoms among Peruvian adult residents amidst a National Lockdown during the COVID-19 pandemic
Source: BMC Psychiatry. 2021 Feb 18;21:111. doi: 10.1186/s12888-021-03107-3 (PMC7890781; doi:10.1186/s12888-021-03107-3)
Supplement: Supplementary file 3 — Additional file 3: Supplementary File 3. PHQ-9 and WASSS items. [file 12888_2021_3107_MOESM3_ESM.docx]

## **Supplementary file 3: PHQ-9 and WASSS items**

| **Patient Health Questionaire-9**  **How often have they been bothered by the following over the past two weeks?** | | |
| --- | --- | --- |
| **N** | **Item** | **Related concept** |
| 1 | Little interest or pleasure in doing things | Anhedonia |
| 2 | Feeling down, depressed, or hopeless | Depressive mood |
| 3 | Trouble falling/staying asleep, sleeping too much | Sleeping problems |
| 4 | Feeling tired or having little energy | Low energy |
| 5 | Poor appetite or overeating | Appetite change |
| 6 | Trouble concentrating on things | Concentration difficulties |
| 7 | Moving or speaking so slowly that other people could have noticed. Or the opposite; being so fidgety or restless that you have been moving around a lot more than usual | Psychomotor problems |
| 8 | Thoughts that you would be better off dead or of hurting yourself in some way. | Suicidal ideation |
| 9 | Feeling bad about yourself or that you are a failure or have let yourself or your family down | Low-self esteem |

| **WHO-UNHCR Assessment Schedule of Serious Symptoms in Humanitarian Settings (WASSS)**  How often during the last two weeks… | | |
| --- | --- | --- |
| **N** | **Item** | **Related concept** |
| 1 | did you feel so afraid that nothing could calm you down — would you say all of the time, most of the time, some of the time, a little of the time, or none of the time? | Fear |
| 2 | did you feel so angry that you felt out of control — would you say all of the time, most of the time, some of the time, a little of the time, or none of the time? | Anger |
| 3 | did you feel so uninterested in things that you used to like, that you did not want to do anything at all? | Uninterested |
| 4 | did you feel so hopeless that you did not want to carry on living? | Hopelessness |
| 5 | did you feel so severely upset about the emergency/disaster/war or another event in your life, that you tried to avoid places, people, conversations or activities that reminded you of such event? | Avoid memories triggers |
| 6 | were you unable to carry out essential activities for daily living because of these feelings? | Impaired functioning |
