# Supplementary material for: Depressive symptoms among Peruvian adult residents amidst a National Lockdown during the COVID-19 pandemic
Source: BMC Psychiatry. 2021 Feb 18;21:111. doi: 10.1186/s12888-021-03107-3 (PMC7890781; doi:10.1186/s12888-021-03107-3)
Supplement: Supplementary file 4 — Additional file 4: Supplementary File 4. Variables values in the analytical sample and Peruvian Census 2017* used for raking. [file 12888_2021_3107_MOESM4_ESM.docx]

## **Supplementary file 4: Variables values in the analytical sample and Peruvian Census 2017* used for raking.**

|  | Census 2017 | | Sample | |
| --- | --- | --- | --- | --- |
|  | **n** | **%** | **n** | **%** |
| **Age group** |  |  |  |  |
| 18-24 | 3,480,936 | 17.25 | 19,563 | 34.05 |
| 25-34 | 4,643,422 | 23.01 | 21,519 | 37.46 |
| 35-44 | 4,075,336 | 20.2 | 10,202 | 17.76 |
| 45-54 | 3,210,942 | 15.91 | 4,016 | 6.99 |
| 55+ | 4,766,919 | 23.62 | 2,146 | 3.74 |
| **Subtotal** | 20,177,555 |  | 57,446 |  |
|  |  |  |  |  |
| **Sex** |  |  |  |  |
| Male | 9,780,154 | 48.47 | 19,386 | 33.75 |
| Female | 10,397,401 | 51.53 | 38,060 | 66.25 |
| **Subtotal** |  |  | 57,446 |  |
|  |  |  |  |  |
| **Education** |  |  |  |  |
| Secondary incomplete or less | 5,193,050 | 25.74 | 2,247 | 3.91 |
| Secondary complete | 10,245,289 | 50.78 | 28,171 | 49.04 |
| Undergraduate or more | 4,739,216 | 23.49 | 27,028 | 47.05 |
| **Subtotal** |  |  | 57,446 |  |
|  |  |  |  |  |
| **Region** |  |  |  |  |
| Lima | 7,005,894 | 34.72 | 32,927 | 57.32 |
| Resto Costa | 4,938,841 | 24.48 | 11,778 | 20.50 |
| Andes | 6,578,264 | 32.6 | 10,762 | 18.73 |
| Selva | 1,654,556 | 8.2 | 1,979 | 3.44 |
| **Subtotal** |  |  | 57,446 |  |
|  |  |  |  |  |
| **Total** | 20,177,555 | 100 | 57,446 | 100 |

*****Data for all adults (≥18 years) who participated in the National census.

**Raking using trimming options for the upper bound of 3500
